# Supplementary material for: MetaRibo-Seq measures translation in microbiomes
Source: Nat Commun. 2020 Jun 29;11:3268. doi: 10.1038/s41467-020-17081-z (PMC7324362; doi:10.1038/s41467-020-17081-z)
Supplement: Supplementary file 10 — Supplementary Data 7 [file 41467_2020_17081_MOESM10_ESM.zip › File2/Confidence_High_Taxonomy/164402_out.krona.html]

Javascript must be enabled to view this page.

members
magnitude
magnitudeUnassigned
count
unassigned
taxon
rank

164402\_out

5

2
superkingdom
5

976
5
phylum

class
5
200643

5
order
171549

5
family
171552

genus
5
838

165179

SRS024132\_contig\_number\_40530SRS103987\_contig\_number\_contig-100\_5978.47678SRS104036\_contig\_number\_8109SRS144506\_contig\_number\_contig-100\_4522.311194SRS144603\_contig\_number\_contig-100\_5772.87194
5
species
